# List of Cases Treated in the Surgical Wards of the Pennsylvania Hospital, and Discharged between November 14th and 28th, 1838

**Published:** 1838-12-05

**Authors:** Henry H. Smith

**Affiliations:** Resident Surgeon


### CLINICAL REPORTS.

### PENNSYLVANIA HOSPITAL.

*List of Cases treated in the Surgical Wards of the Pennsylvania Hospital, and discharged between November 14th and 28th,* 1838. Dr. T. Harris, Attending Surgeon.

[Reported by Henry H. Smith, M.D., Resident Surgeon.]

A case of slight contusion of the thigh, from the kick of a horse, was admitted November 12th; treated by perfect rest and frictions, and discharged at his own request, November 14th.

A case of fracture of the lower end of the radius, a short distance above the wrist joint, was admitted October 17th. It was dressed in the usual manner, with two padded splints, one on the back and the other on the front of the arm, extending from beyond the fingers to the elbow, loosely bandaged for the first three days, afterwards more firmly. Discharged cured, with perfect motion of all the joints, November 17th, thirty-one days after the accident.

A case of fracture of the neck of the scapula, caused by a fall from the fourth story of a house, on a projecting plank, was admitted October 27th three hours after the accident. On his admission it appeared to be a dislocation of the head of the humerus forwards, but its immediate reduction on carrying the elbow upwards and backwards and the crepitus showed the true injury. Violent inflammation around the joint followed. The limb was, therefore, simply suspended in a handkerchief, the patient confined to bed, leeched, and lead-water cloths kept constantly to the part. This reducing the inflammation, the sling, collar, and pad of the clavicle apparatus were applied, and reduced the parts nearly to their proper position. This dressing was continued three weeks, when a common sling was substituted. The man was discharged November 28th, thirty-two days after the accident, with the perfect use of the limb, but with a little deformity, caused by the bone being drawn forwards by the great action of his muscles.

A case of violent twisting of the muscles of the neck, caused by a fall, was admitted November 16th. On admission there was reason to suppose a partial dislocation had occurred, as the arm and part of the body of one side were paralysed; the patient was also unable to turn the head, or move it forwards or backwards. An examination of the spinous processes, however, showed that there was no displacement, and, by raising the head, it was possible to turn it partially. Cups and frictions were applied, the patient placed in an easy position, not being able to lie down, and the neck surrounded by raw cotton. These means afforded relief in the course of thirty-six hours, and the patient was discharged with perfect command of the neck, November 23d.

A case of contusion of the deltoid muscle, caused by a fall, was admitted October 24th; there was great swelling and perfect inability to move the arm. The patient was placed in bed, cups applied to the part, afterwards leeches, and warm cloths. This relieving the inflammation, the arm was placed in a sling, and the man allowed to walk about. The muscle wasted considerably, and there was perfect inability to raise the arm or to move it at all. Frictions of soap liniment were afterwards used, and he was discharged with tolerable use of his arm, at his own request, November 21st, twenty-eight days after the accident.

This case offers an instance of an interesting class of injuries, viz. contusion and subsequent paralysis of the muscles. The patient was remarkably muscular on admission, yet before his discharge the shoulder had the flat appearance seen after a dislocation, and he had regained but partial use of the limb, although twenty-eight days had elapsed since the accident.

A case of compound fracture of the skull, caused by an axe falling on the head, was admitted July 29th, 1838. There was an incised wound of the scalp, two and a half inches long, and a simple fissure in the cranium without depression. The man was perfectly sensible and walked into the house. The wound was drawn together and dressed with lint, exfoliation of the bone afterwards took place, and continued at intervals until October 28th. Erysipelas attacked the scalp on November 15th, and the man was discharged cured, November 21st, one hundred and fifteen days after the accident.

A case of compound fracture of the skull, caused by the kick of a horse, was admitted 22d of October; hernia cerebri followed, and death November 23d. Reported at length in this number.

A case of paronychia of the thumb, in a girl, was admitted October 24th; the parts were freely opened; the last phalanx came away, and she was discharged November 26th, thirty-three days after admission.

A second case of the same, in a man, was admitted October 10th; treated by free incisions, &c., loss of the bone followed, and the patient was discharged November 28th, forty-nine days after his entrance.

A case of fracture of both bones of the leg, was admitted October 1st; treated in the usual manner, and discharged November 28th, fifty-eight days after admission.

A case of simple transverse fracture of the tibia, was admitted October 28th; kept in a fracture box for two weeks, afterwards dressed with the pasteboard splints, and allowed to walk about. Discharged November 28th, four weeks after the accident; union perfectly firm, and the patient able to move with a stick.

A case of fracture of the tibia with bending of the fibula, consequent on a fall, in a boy, eleven years old, suffering under mollities ossium, was admitted September 3d. The limb was kept in the fracture box; union being slow, blisters were applied constantly for three weeks, when it became firm; pasteboard splints were applied November 18th, and the boy allowed to walk. He was discharged cured, November 28th, eighty-six days after the accident.

This was the sixth time this boy had been in the institution within the last five years, the other leg having been broken twice.

